# Histopathological Characterization of Cases of Spontaneous Fatal Feline Severe Fever with Thrombocytopenia Syndrome, Japan

**DOI:** 10.3201/eid2704.204148

**Published:** 2021-04

**Authors:** Yusuke Sakai, Yuko Kuwabara, Keita Ishijima, Saya Kagimoto, Serina Mura, Kango Tatemoto, Ryusei Kuwata, Kenzo Yonemitsu, Shohei Minami, Yudai Kuroda, Kenji Baba, Masaru Okuda, Hiroshi Shimoda, Masashi Sakurai, Masahiro Morimoto, Ken Maeda

**Affiliations:** Yamaguchi University, Yamaguchi, Japan

**Keywords:** Severe fever with thrombocytopenia syndrome virus, SFTS, SFTSV, zoonoses, histopathology, cats, felines, viruses, vector-borne infections, tickborne diseases, Japan

## Abstract

Severe fever with thrombocytopenia syndrome (SFTS) is an emerging tickborne infectious disease caused by SFTS virus (SFTSV). We report 7 cases of spontaneous fatal SFTS in felines. Necropsies revealed characteristic lesions, including necrotizing lymphadenitis in 5 cases and necrotizing splenitis and SFTSV-positive blastic lymphocytes in all cases. We detected hemorrhagic lesions in the gastrointestinal tract in 6 cases and lungs in 3 cases, suggesting a more severe clinical course of SFTS in felids than in humans. We noted necrotic or ulcerative foci in the gastrointestinal tract in 3 cases, the lung in 2 cases, and the liver in 4 cases. We clarified that blastic lymphocytes are predominant targets of SFTSV and involved in induction of necrotic foci. We also found that thymic epithelial cells were additional targets of SFTSV. These results provide insights for diagnosing feline SFTS during pathological examination and demonstrate the similarity of feline and human SFTS cases.

Severe fever with thrombocytopenia syndrome (SFTS) is an emerging infectious disease characterized by acute onset of high fever, hemorrhagic tendency, gastrointestinal and neurologic symptoms, thrombocytopenia, and leukocytopenia (*1*–*4*). The causative agent of SFTS is a novel *Dabie bandavirus*, SFTS virus (SFTSV), of the family *Phenuiviridae* (*5*). On the basis of epidemiologic evidence, SFTS is classified as a tickborne disease, and the main reservoir and vector involving human infection is thought to be the *Haemaphysalis longicornis* tick (*6*,*7*). In addition, various species of domestic and wild mammals, including goats, sheep, cattle, pigs, dogs, cats, boars, and deer, have been found to harbor SFTSV genomic RNA or SFTSV antibodies (*8*–*10*). These data demonstrate the infectivity of SFTSV in these animal species and circulation of SFTSV between ticks and animals in nature. Reported cases of SFTS in cheetahs and domestic cats have shown that nonhuman mammalian species can develop fatal disease similar to human SFTS (*11*,*12*). Susceptibility of cats to SFTSV also was confirmed by experimental SFTSV infection in cats, which caused a high incidence of severe hemorrhagic fever (*13*). Analyses of these cases confirmed shedding of viral particles from saliva and feces (*11*,*13*), which can cause transmission of SFTSV from diseased animals to humans (*14*). 

Analysis of animals with SFTSV infection can inform the pathogenesis of SFTS in humans. Experimental infection in wild type and α/β interferon receptor knockout mice, rhesus macaques (*Macaca mulatta*), and signal transducer and activator of transcription-2 knockout hamsters have been reported (*15*–*19*), but hepatitis and splenitis have been reproduced only in hamster models (*19*). However, hemorrhagic and necrotic lesions in the liver, spleen, intestines, and lymph nodes have been reported only in fatal SFTS cases in humans and experimentally infected felines (*13*,*20*–*22*). Investigations of disease in animals that mimics human SFTS is crucial for informing prevention of animal-to-human transmission and controlling virus transmission among animals and ticks. Analysis of fatal cases in felines can clarify the pathology of SFTS and inform STFS diagnosis in animals. We provide evidence of characteristic macroscopic and microscopic lesions collected from 7 cases of spontaneous fatal SFTS in felines.

## Materials and Methods

### Histology

We performed necropsies on 7 cats with SFTS symptoms, such as acute onset of thrombocytopenia, leukocytopenia, and lethargy (Table 1). We confirmed SFTSV infection by conventional reverse transcription PCR (RT-PCR) using 2 primer pairs targeting the small segment of the SFTSV genome (*23*). We collected and fixed tissue samples in 10% neutral buffered formalin and then processed the samples to create paraffin-embedded tissue sections. We cut tissue into sections 4-μm thick and stained sections with hematoxylin and eosin for histopathologic examination.

**Table 1:** Clinicopathological findings in 7 cats with fatal severe fever with thrombocytopenia syndrome, Japan*

Clinical findings	Case no.						
	1	2	3	4	5	6	7
Clinical signs							
Anorexia	Y	Y	Y	Y	Y	Y	Y
Lethargy	Y	Y	Y	Y	Y	Y	Y
Neurologic signs	N	N	N	Y	N	N	N
Vomiting	N	Y	N	N	N	Y	N
Body temperature, °C	39.4	NA	NA	NA	39.3	39.9	39
Erythrocytes, 10^4^ cells/μL	546	688	NA	364	718	820	593
Leukocytes, cells/μL	3,080	800	3,000	190	700	2,500	1,290
Platelets, cells/μL	38,000	7,000	0	0	<11,000	52,000	0
ALT, IU/L	105	NA	476	141	331	NA	58
AST, IU/L	51	NA	>1,000	4	1,010	NA	NA
ALP, IU/L	188	NA	NA	NA	NA	NA	<10
Total bilirubin, mg/dL	4.4	5.8	4.8	5.7	NA	NA	9.3
CPK, IU/L	NA	373	>2,000	>2,000	1,444	NA	NA
*ALP, alkaline phosphatase; ALT, alanine aminotransferase; AST, aspartate aminotransferase; CPK, creatine phosphokinase; NA, not available.							

### Immunohistochemistry

We subjected the 4-μm thick tissue sections to immunohistochemical staining. After deparaffinization, we performed antigen retrieval by incubating sections in 0.1% trypsin at 37°C for 20 min to obtain immunoglobulin (Ig) lambda chain; or by heating at 121°C for 5 min in pH 6.0 citrate buffer for SFTSV and CD3 staining; or pH 9.0 Tris-EDTA buffer for CD79a and Ki67 staining. After washing with phosphate-buffered saline (PBS), we inactivated endogenous peroxidase by immersion in 3% hydrogen peroxide in PBS. After treatment with 5% bovine serum albumin in PBS for 30 min, we incubated the sections with rabbit polyclonal anti-SFTSV antibody (diluted 1:1,000; gift from Shigeru Morikawa, Okayama University of Science, Okayama, Japan); FLEX Polyclonal Rabbit Anti-Human CD3 Ready-to-Use antibody (Dako, https://www.agilent.com); Monoclonal Mouse Anti-Human CD79a Antibody Clone HM57 (diluted 1:50; Dako); Mouse Monoclonal Anti-Ki67 Clone MIB-1 (diluted 1:1,000; eBioscience, https://www.thermofisher.com); or Polyclonal Rabbit Anti-Human Ig Lambda Light Chains (diluted 1:100; Dako). After washing with PBS, we incubated the sections with EnVision+/HRP Rabbit (Dako) horseradish peroxidase (HRP)–labeled polymer anti-rabbit or EnVision+/HRP Mouse (Dako) HRP-labeled polymer anti-mouse. We then visualized positive signals by peroxidase-diaminobenzidine reaction, and counterstained sections with hematoxylin stain.

### Immunofluorescence

We performed double immunofluorescence labeling with cytokeratin-SFTSV and CD204-SFTSV on 4-μm thick tissue sections. We performed heat-mediated antigen retrieval for cytokeratin-SFTSV in pH 6.0 citrate buffer and for CD204-SFTSV antigen pH 9.0 Tris-EDTA buffer. After washing with PBS and blocking with 5% bovine serum albumin, we incubated the sections for 1 h at room temperature with a mixture of rabbit polyclonal anti-SFTSV antibody (diluted 1:1,000; TransGenic Inc., https://www.transgenic.co.jp) and mouse monoclonal anti-CD204 (diluted 1:400; TransGenic Inc.) or mouse monoclonal anti-cytokeratin clone AE1/AE3 (diluted 1:200; Dako). After washing with PBS, we incubated the sections in a mixture of Alexa Fluor 488 anti-rabbit IgG (diluted 1:400; Abcam, https://www.abcam.com), Alexa Fluor 594 anti-mouse IgG (diluted 1:400; Abcam), and DAPI (Dojindo, https://www.dojindo.com). We then analyzed the tissue sections by using an LSM 710 (Leica, https://www.leicabiosystems.com) confocal microscope.

## Results

### Gross Findings

**Figure 1:**
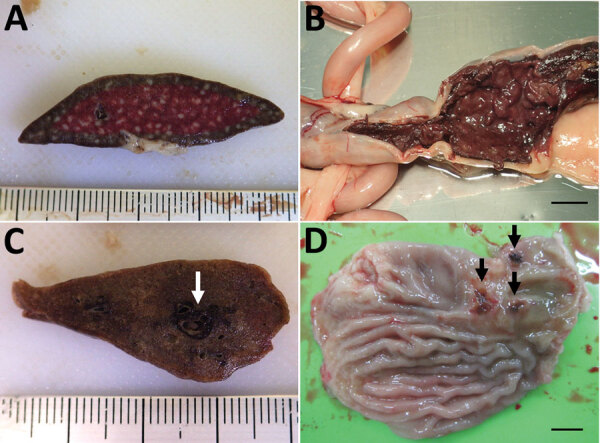
Gross pathology of lesions from cats with fatal severe fever with thrombocytopenia syndrome, Japan. A) Enlarged follicles (white spots) in the spleen. Ruler represents centimeters. B) Hemorrhage in the colon. Scale bar indicates 1 cm. C) Hemorrhage in the lung; white arrow indicates pulmonary hemorrhage around the trachea. Ruler represents centimeters. D) Gastrointestinal ulcers (black arrows) were also seen in some cases. Scale bar indicates 1 cm.

Among the 7 cats with SFTS, gross lesions typically were characterized by changes in the lymphoid organs and hemorrhage (Table 2). In all the cases, we noted red enlarged lymph nodes from various regions. Although splenomegaly was unclear to mild, we noticed enlarged follicles appearing as multiple white spots in the spleen (Figure 1, panel A) in all cats. We detected hemorrhagic lesions in the gastrointestinal tract in 6 cats (Figure 1, panel B) and in the tracheal region of the lungs in 3 cats (Figure 1, panel C). In 3 cats, gastrointestinal lesions resulted in grossly obvious ulcers (Figure 1, panel D). Jaundice was detected in 5 cats.

**Table 2:** Gross lesions in 7 cats with fatal severe fever with thrombocytopenia syndrome, Japan

Lesions	Cases, no. (%)
Enteric hemorrhage	6 (85.7)
Gastrointestinal ulcer	3 (42.8)
Pulmonary hemorrhage	3 (42.8)
Jaundice	5 (71.4)

### Lymphatic System Lesions

**Figure 2:**
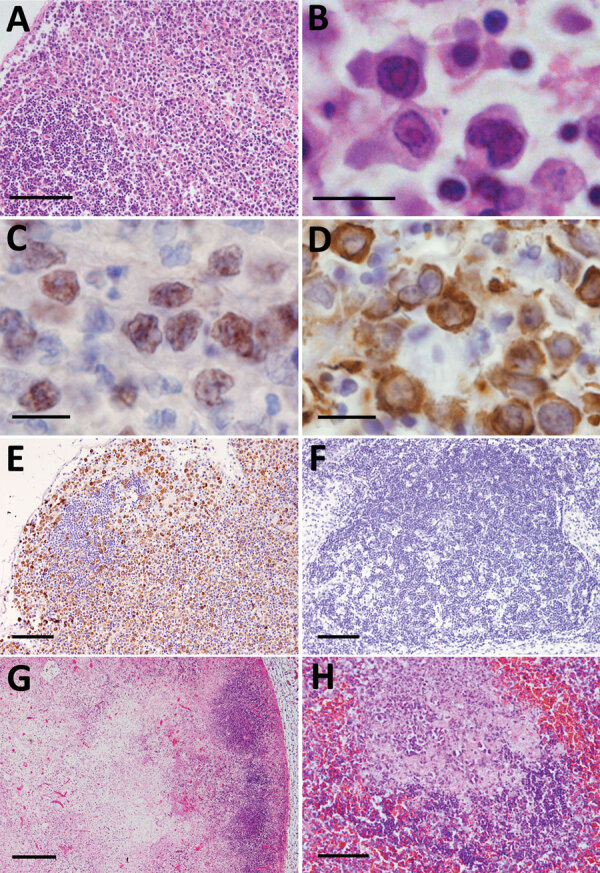
Histopathological lesions in the lymphoid organs from fatal cases of severe fever with thrombocytopenia syndrome (SFTS) in cats, Japan. A) Hematoxylin & eosin (HE)–stained lymph node demonstrating accumulation of blastic lymphocytes around the lymphoid follicle. Scale bar indicates 100 μm. B) HE-stained blastic lymphocytes from the lymph nodes demonstrating highly pleomorphic cells with large clear nuclei and prominent nucleoli, resembling immunoblasts. Scale bar indicates 10 μm. C, D) CD79a-stained (C) and immunohistochemistry-stained (D) blastic lymphocytes from the lymph nodes. Scale bar indicates 10 μm. E) Lymph node stained by immunohistochemistry revealing SFTS virus–positive blastic lymphocytes distributed around the follicle. Scale ar indicates 100 μm. F) Immunohistochemistry-stained hyperplastic lymph node demonstrating no SFTSV-positive cells or necrotic foci. Scale bar indicates 100 μm. G) Necrotic lymphadenitis in HE-stained lymph node. Scale bar indicates 200 μm. H) HE-stained spleen demonstrating necrotic lesions in the splenic follicle. Scale bar indicates 50 μm.

Histologically, characteristic SFTS lesions were observed in the lymphoid organs, such as the lymph nodes, spleen, and Peyer’s patches. We noted lesions in the collected lymph nodes in all 7 cats (Table 3). In the cortex of SFTSV-affected lymph nodes, we observed an accumulation of the large blastic lymphocytes, as those described in a human case (*21*). The blastic lymphocyte cells were morphologically characterized by large, clear irregular-shaped nuclei with prominent central nucleoli and were similar to the morphology of the immature activated-B cells, called immunoblasts (Figure 2, panels A, B). Compatible with immunoblast-like cell morphology, these cells were considered cells of the B cell lineage because they were positive for CD79a expression (Figure 2, panel C). Immunohistochemistry also revealed that SFTSV antigens were exclusively detected in these blastic lymphocytes and SFTSV-positive blastic lymphocytes distributed in the cortex and paracortex area surrounding lymphoid follicles (Figure 2, panels D, E). Regardless of enlargement, some lymph nodes had neither SFTSV-positive cells nor necrotic lesions and were simply diagnosed as hyperplastic lymph nodes (Table 3; Figure 2, panel F). In some lymph nodes, the lesions proceeded to necrotizing lymphadenitis with SFTSV-positive blastic lymphocytes (Figure 2, panel G). In all cases, we detected SFTSV-positive blastic lymphocytes and necrotic foci in the spleen, mainly in the follicular area (Figure 2, panel H). We collected the thymus glands from 4 cats and observed infiltration of SFTSV-positive blastic lymphocytes mainly in the cortices of all specimens. Hemorrhagic and necrotic lesions also were observed in the thymus glands. As reported in humans (*20*–*22*), we observed numerous hemophagocytic macrophages in the lymphoid organs of all cases.

**Table 3:** Lesions in the lymphatic system from 7 cats with fatal severe fever with thrombocytopenia syndrome, Japan*

Lesions	Case no., n = no. lymph nodes assessed						
	1, n = 4	2, n = 1	3, n = 4	4, n = 9	5, n = 7	6, n = 4	7, n = 6
Hyperplasia without SFTSV-positive cells	0	0	3	0	2	2	0
SFTSV-positive blastic lymphocytes	3	1	1	6	2	1	2
Necrotizing lymphadenitis	1	0	0	3	3	1	4
*SFTSV, severe fever with thrombocytopenia syndrome virus.							

### Intestinal Tract Lesions

**Figure 3:**
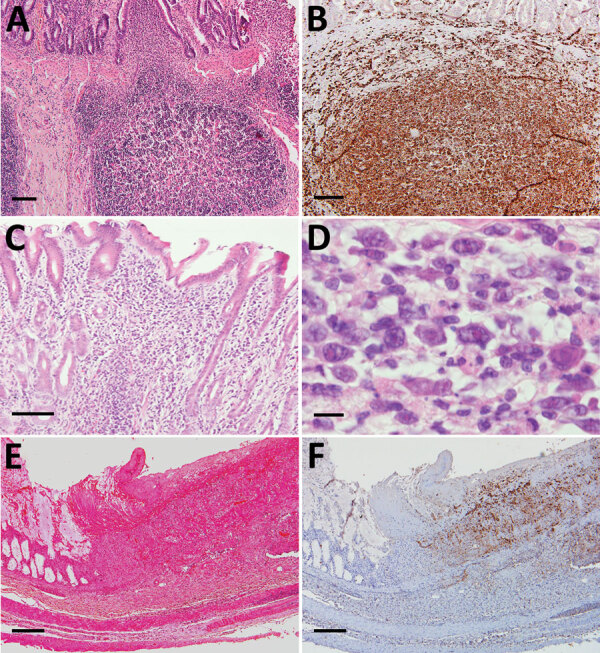
Histopathological lesions in the intestinal tracts from fatal cases of severe fever with thrombocytopenia syndrome (SFTS) in cats, Japan. A, B) Hematoxylin & eosin (HE)–stained (B) and immunohistochemistry-stained (A) ileum sections demonstrating enlargement of Peyer’s patch and accumulation of SFTSV-positive blastic lymphocytes. Scale bars indicates 100 μm. C) HE-stained colon sections demonstrating infiltration of lymphocytes into the lamina propria. Scale bar indicates 100 μm. D) High power magnification of panel C demonstrating the infiltrating lymphocytes were blastic lymphocytes. Scale bar indicates 10 μm. E, F) HE stained (E) and immunohistochemistry-stained (F) ulcerative lesions in the cecum. Scale bars indicate 200 μm.

We noted SFTSV-positive blastic lymphocytes in the intestinal tract in all cases (Figure 3), mainly in the Peyer’s patches, the localized lymphoid follicular structure in the intestinal submucosa; some of these cells infiltrated the lamina propria (Figure 3, panels A, B). We observed infiltration of these cells in all hemorrhage lesions and ulcers (Figure 3, panels C, D), suggesting a relationship between blastic lymphocytes and lesions.

### Liver and Lung Lesions

**Figure 4:**
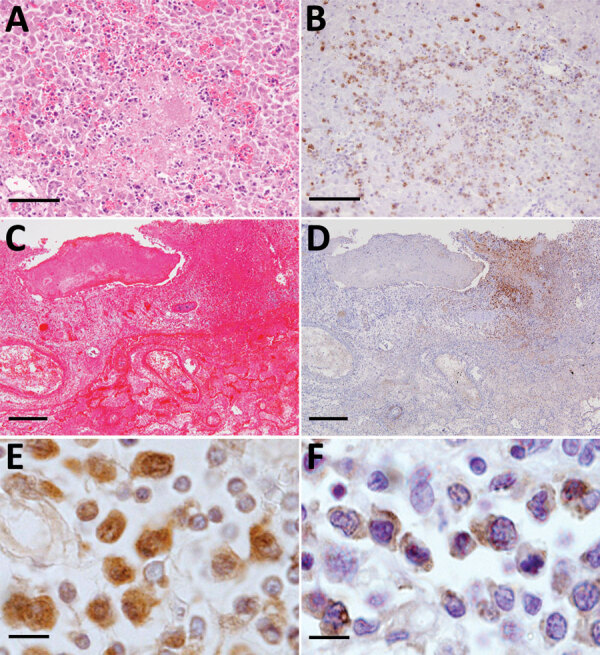
Necrotic foci in the liver and lung from fatal cases of severe fever with thrombocytopenia syndrome (SFTS) in cats, Japan. A, B) Hematoxylin & eosin (HE)–stained (A) and immunohistochemistry-stained (B) liver sections demonstrating SFTS virus–positive blastic lymphocytes in the necrotic foci. Scale bars indicate 100 μm. C, D) HE-stained (C) and immunohistochemistry-stained (D) lung sections demonstrating lymphocytes in the necrotic foci from the lungs. Scale bars indicates 200 μm. E, F) Ki67 (E) and Ig lambda chain (F) immunohistochemistry positively staining blastic lymphocytes. Scale bars indicates 10 μm.

In the livers from 3 cats, we noted formation of small necrotic foci (Figure 4, panel A). The necrotic lesions always were surrounded by SFTSV-positive blastic lymphocytes (Figure 4, panel B), but the cells usually were distributed in the portal area. We also found bile pigmentation and hemophagocytic macrophages in the liver. 

Feline SFTS pulmonary lesions consisted of hemorrhage in 3 cases and formation of necrotic foci in the interstitial tissues surrounding the trachea in 2 cases (Figure 4, panel C). Like hepatic necrotic foci, the pulmonary necrotic foci we observed in these cases also showed SFTSV-positive blastic lymphocytes (Figure 4, panel D).

### SFTSV-Positive Blastic Lymphocyte Characterization

Immunohistochemistry revealed that SFTSV-positive signals were limited to the blastic lymphocytes, and these were of B cell lineage and expressed CD79a (Figure 2, panel C). We performed further characterization by using immunohistochemical stains against Ig lambda chain and Ki67. The results demonstrated that SFTSV-positive atypical lymphocytes expressed Ig lambda chain and Ki67 (Figure 4, panels E, F). Hence, we considered these cells plasmablasts, which are immature plasma cells retaining proliferation activities.

**Figure 5:**
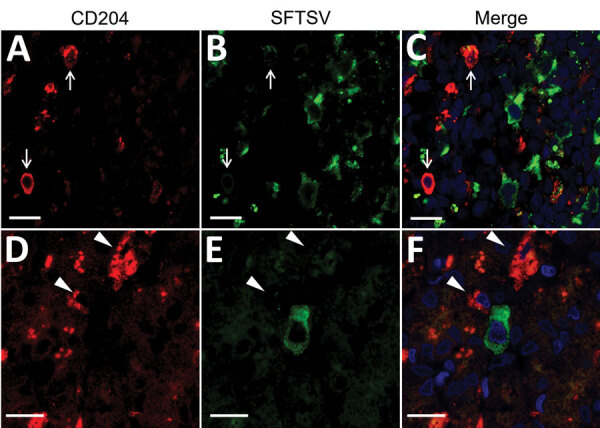
Double-labeling immunofluorescent staining of the lymph node (A–C) and the liver (D–F) from fatal cases severe fever with thrombocytopenia syndrome (SFTS) in cats, Japan. Red indicates signals of CD204. Green indicates signals of SFTS virus. Blue indicates nuclei labeled with DAPI. Arrows in panels A–C indicate CD204-positive macrophages in the lymph node. Arrows in panels D–F indicate CD204-positive kupffer cells in the liver. Scale bars indicate 10 μm.

The presence of SFTSV antigens in macrophages has been reported (*15*,*24*). To investigate whether macrophages in our cases consisted of SFTSV-positive cells along with B cells, we performed double immunofluorescence analysis of the macrophage markers CD204 and SFTSV. The results demonstrated that SFTSV-positive cells were not CD204-positive (Figure 5).

### SFTSV-Positive Cells in the Thymus

**Figure 6:**
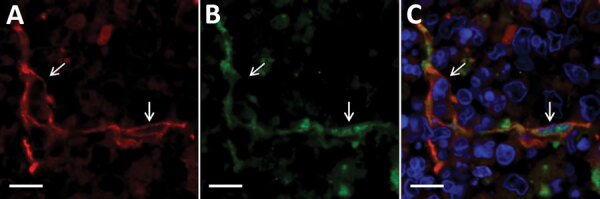
Double-labeling immunofluorescent staining of the thymus from fatal cases severe fever with thrombocytopenia syndrome (SFTS) in cats, Japan. Arrows indicate thymic epithelial cells. A, D) Red indicates signals of cytokeratin. B, E) Green indicates signals of SFTS virus. C, F) Blue indicates nuclei labeled with DAPI. Scale bars indicate 10 μm.

In addition to blastic lymphocytes, we found SFTSV-positive cells with spindle-to-polygonal morphology in the thymus gland. Double immunofluorescence analysis of SFTSV and cytokeratin revealed that these cells were thymic epithelial cells (Figure 6).

## Discussion

We analyzed the pathological changes in 7 fatal cases of SFTS in domestic felids. We detected characteristic lesions in the lymphoid organs, including the lymph nodes and spleen. We observed gross enlargement and hemorrhage of multiple lymph nodes and formation of white spots in the spleen in all 7 cases (Figure 1, panel D). Because these findings are highly suggestive of but not specific for feline SFTS, additional cases with these findings should be further examined to confirm viral infection by testing, such as RT-PCR.

Similar to human cases (*20*–*22*), we frequently observed necrotic lymphadenitis in this study and found accumulation of SFTSV-positive atypical lymphocytes in all 7 cases (Table 2). In addition, all 7 cats had necrotic splenitis, similar to human SFTS cases (*20*,*21*). Therefore, lesions in the lymphoid organs, or accumulation of atypical B cells can be considered characteristic and highly specific for lesions in SFTS for both humans and felines. Furthermore, these lesions can be useful indicators of whether experimental infection in laboratory animals appropriately reproduces spontaneous SFTS. In fact, feline cases of experimental infection showed necrotic lymphadenitis and splenitis (*13*).

Although some reports have detected SFTSV antigens in macrophages, our study demonstrated that SFTSV-positive cells mostly were blastic lymphocytes (Figure 2, panels B–D; Figure 4, panels E, F). Immunofluorescence revealed that only punctate signals were detected in the macrophages (Figure 5). This result indicates that macrophages can phagocytose SFTSV but do not support SFTSV replication. Our findings also showed that SFTSV targets the cells of B-cell lineage and that plasmablasts were the predominant site of viral replication. Such tropism of SFTSV to plasmablast also has been reported in human cases and in vitro analysis clarified SFTSV targeted plasmablastic cell line, not B cell lymphoma cell lines (*25*). However, why large numbers of plasmablasts appeared and accumulated in the lymph nodes remains unclear. Dysregulated immunological response of plasmablasts to SFTSV infection and viral modulation of host-plasmablast dynamics are 2 possible causes of plasmablast accumulation in the lymph nodes. Furthermore, in all cases, we found SFTSV-positive blastic lymphocytes in and around the intestinal ulcerative lesions (Figure 3, panel F) and necrotic foci in the liver and lungs (Figure 4, panels A–D), suggesting a role of blastic lymphocytes in the pathogenesis of ulcers and necrotic lesions. Depositing of Ig in the necrotic foci and expression of death ligands in SFTSV-positive cells should be analyzed in future cases. Expression of cell death-inducing factors on SFTSV-positive blastic lymphocytes, such as self-reactive Ig and death ligands, could indicate a relationship between these cells and necrotic foci; further study is warranted. Our study demonstrated that the thymic epithelial cells can be another target of SFTSV. However, the significance of viral infection in the thymic epithelium is unclear.

Gastrointestinal manifestation is one of the clinical features of human SFTS (*1*–*4*). A lethal case of severe intestinal hemorrhage and another case of multiple gastrointestinal ulceration have been reported in humans (*20*,*26*). However, the incidence of these lesions is unclear because cases without obvious intestinal findings also have been reported (*21*). Experimental SFTSV infection in cats and our results have demonstrated a high rate of gastrointestinal hemorrhage (6/7 cases; Figure 1, panel A) and gastrointestinal ulcers (3/7 cases; Figure 3, panel E), which suggests that human and feline cases share a common pathogenesis and that feline cases show more severe gastrointestinal lesions than human cases (*13*). Pulmonary hemorrhage and necrotic foci also suggest the severity of feline SFTS. This severe pathogenesis in many tissues can cause high lethality, ≈70% (*27*), suggesting that feline SFTS is a typical lethal viral hemorrhagic fever.

Jaundice was frequently observed in our cases (Table 2) and in experimental infection in cats (*13*). Also, marked elevation of serum hepatic enzymes were detected in most cases (Table 1). However, in this study, morphologic lesions in the liver were only sporadic small necrotic foci, insufficient to cause systemic jaundice and marked elevation of serum hepatic enzymes. Microscopic lesions of the liver were also mild in human SFTS cases and in cats with experimental infection (*13*,*21*). These findings suggest microscopically undetectable hepatic damage or functional failure of the hepatobiliary system. Further analysis is needed to clarify the mechanism of liver damage in SFTS.

Other typical clinical manifestations of SFTS in humans include neurologic signs, thrombocytopenia, and leukocytopenia (*1*–*4*). Although neurologic signs were unclear in the cats in our study, thrombocytopenia and leukocytopenia were clinically detected. Analysis of the bone marrow and central nervous system in feline SFTS cases will help clarify pathogenesis.

Our study demonstrated typical lesions of spontaneous fatal cases of feline SFTS, consisting of similar pathological lesions and a more severe hemorrhagic tendency than in human SFTS cases. Information on disease in animals that mimics human SFTS can help prevent animal-to-human transmission. Thus, we believe feline models can be used to study the pathogenesis of SFTS.
